# Modulating the Activity of Ventromedial Prefrontal Cortex by Anodal tDCS Enhances the Trustee’s Repayment through Altruism

**DOI:** 10.3389/fpsyg.2016.01437

**Published:** 2016-09-22

**Authors:** Haoli Zheng, Daqiang Huang, Shu Chen, Siqi Wang, Wenmin Guo, Jun Luo, Hang Ye, Yefeng Chen

**Affiliations:** ^1^College of Economics and Interdisciplinary Center for Social Sciences, Zhejiang University, HangzhouChina; ^2^School of Economics, Center for Economic Behavior and Decision-making, Neuro and Behavior EconLab, Zhejiang University of Finance and Economics, HangzhouChina

**Keywords:** ventromedial prefrontal cortex, transcranial direct current stimulation, trustworthiness, altruism, trust game, dictator game

## Abstract

Trust and trustworthiness are essential to an efficient economy and play crucial roles in social life. Previous evidence from behavioral experiments has revealed that the trustworthiness of individuals is closely related with their altruistic preference. It has been demonstrated that the ventromedial prefrontal cortex (vmPFC) is associated with decisions involving trustworthiness. Moreover, vmPFC lesion patients showed less trustworthiness and altruism than control subjects, indicating the indispensable role of this specific brain area in human social interactions. However, the causal relationship between this neural area and trustworthiness, as well as altruism, has not been fully revealed. The potential neural basis behind the behavior of trustees’ repayment has also seldom been discussed. In the present study, we aimed to provide evidence of a direct link between the neural and behavioral results through the application of transcranial direct current stimulation (tDCS) over the vmPFC of our participants. We found that activating the vmPFC could promote both the trustworthiness and altruism of our participants. We also show that enhancing the excitability of the vmPFC using tDCS increased the trustworthiness of the participants, and this promoting effect might be attributable to the enhancement of individuals’ altruistic preference. In addition, we revealed that the enhancing effect in trustworthiness and altruism might be specific to the activation of the vmPFC by applying tDCS over another brain region within the prefrontal cortex as a control site. Crucially, our findings provide direct evidence supporting the critical role of the vmPFC in cooperative behaviors in economic interactions, especially the trustees’ repayment in the trust game and the dictators’ altruistic transfer in the dictator game.

## Introduction

“Trust is one of the most important synthetic forces within society” (Simmel and Wolff, 1950). It is well recognized that trust permeates friendship relations, family relations, and economic relations. Interpersonal trust is also a central concept for understanding economic interactions among human beings because it is obvious that the absence of trust among trading partners severely hampers market transactions. Clearly, there are good reasons for social scientists to be interested in the concept of “trust.”

Extensive works by economists have debated the potential forces inducing behaviors of both trust and trustworthiness among individuals who fail to fit the presumption of the *Homo economicus* hypothesis (Berg et al., 1995; Cook and Cooper, 2003; Bohnet and Zeckhauser, 2004; Cox, 2004; Ashraf et al., 2006; Schechter, 2007). Numerous behavioral experiments have focused on the preferences driving the choices in the role of trustor, while relatively few studies have discussed the motives behind the trustee’s repayment. It has been demonstrated that the trustworthiness of trustees could be explained by unconditional preferences such as altruism (Andreoni and Miller, 2002; Holm and Danielson, 2005) and conditional preferences such as reciprocity (Camerer, 2003). In both between-subject and within-subject designs, trustworthiness of individuals was consistently correlated with altruistic preferences of participants (Cox, 2004; Ashraf et al., 2006).

Trustworthiness has been shown to be driven by various motives including altruistic preference, and the relationship between altruism and its neural basis has been demonstrated in neuroscience studies. Functional magnetic resonance imaging (fMRI) studies revealed that neural activity in the medial prefrontal cortex (mPFC) predicted greater empathy and altruistic motivation (Mathur et al., 2010; Waytz et al., 2012). Clinical lesion studies observed that patients with damage to the ventromedial prefrontal cortex (vmPFC) gave significantly less in the dictator game as well as showing less trustworthiness in the trust game, indicating that the vmPFC is indispensable in both altruistic and trustworthy decisions (Krajbich et al., 2009; Moretto et al., 2013).

In accordance with behavioral studies, numerous neuro-science studies have investigated the neural basis of trust behaviors (McCabe et al., 2001; Delgado et al., 2005; King-Casas et al., 2005; Krueger et al., 2007; Tzieropoulos, 2013), while the neural system related to the trustee’s trustworthiness remains largely unknown. Few studies have investigated the trustworthiness of trustees, while the neural bases for factors including reputation, risk, and benefit have been reported (Baumgartner et al., 2009; Knoch et al., 2009; van den Bos et al., 2009). Li et al. (2009) suggested that the trustee’s brain revealed greater activation of areas including the vmPFC, the lateral orbitofrontal cortex, the posterior cingulate cortex, and the right amygdala and indicated that the vmPFC also predicts altruistic behavior. In previous neurocognitive studies, the vmPFC has also been considered critical for valuing social information, and damage to the vmPFC resulted in severe disruption of emotion and contributed to impairments in decision making, planning and behavior regulation (Damasio, 1994; Anderson et al., 2006). More specifically, in a clinical study, Moretto et al. (2013) revealed that patients with lesions to the vmPFC were less trustworthy than control participants. However, these fMRI or lesion case studies have failed to demonstrate a direct causal link between the neural activity in these brain regions and the trustees’ trustworthiness.

In contrast, brain stimulation techniques such as transcranial direct current stimulation (tDCS) that interfere with brain activity non-invasively could establish causal connections between the brain and decisions without many of the confounds inherent to natural lesion studies. Colzato et al. (2015) applied tDCS over vmPFC and found no relationship between interpersonal trust and the vmPFC, while the relationship between vmPFC activity and trustworthiness has not been investigated. More recently, another tDCS study reported that modulating activity in the orbitofrontal cortex changed the trustees’ repayment in a trust game and indicated that their observation was due to the effect of a guilt-related brain area: the orbitofrontal cortex (Wang et al., 2016). However, the participants in their study were not playing with real person as counterparts and the potential preference inducing the changes of the participants’ trustworthiness by tDCS was not revealed in their study.

In the current study, we applied tDCS in which the target electrode was placed over the vmPFC and the reference electrode was placed over visual cortex, which is trust irrelevant according to Colzato et al. (2015). Since visual cortex of the brain is responsible for processing visual information and its location is relatively far from the target brain area, it has been selected as the location for the reference electrode in several previous studies (Colzato et al., 2015; Nihonsugi et al., 2015). The advantage of such a design is that the combined stimulatory influences of the two different brain regions might be eliminated, and any stimulation effects may be due to the excitability of the target brain area (for example, vmPFC). To eliminate our participants’ suspicion of whether they were playing with real person rather than just playing against computer programs, we recruited 10 individuals in the same laboratory during each experimental session who were interacting with anonymous others during the experiment. Moreover, the trustworthiness of our participants was measured by a trust game, and their altruism was measured by a dictator game, which has been demonstrated to be valid in economic interactions. The aim of our study is to determine whether modulating the excitability of the vmPFC can directly influence our participants’ trustworthiness and altruism. After receiving tDCS stimulation (anodal, cathodal, or sham), the participants were required to accomplish decision-making tasks, including a trust game and a dictator game. By comparing repayment rates in the trust game between different tDCS stimulations, a causal relationship between the excitability of the vmPFC and the trustworthiness of the participants might be observed. Furthermore, to test the potential preferences inducing the repayment by our participants, we investigated whether there was any difference between the participants’ altruistic preferences among different stimulation groups. We considered that the observation of similar patterns of significant difference between trustworthiness and altruistic preferences of our participants among different stimulation conditions would demonstrate that modulating the excitability of certain neural areas (such as the vmPFC) might alter the trustworthiness of our participants through its one possible driving force: altruism.

## Experiment 1

### Materials and Methods

#### Subjects

Sixty right-handed healthy subjects (mean age 21.5 years, ranging from 17 to 31 years; 31 females) with no history of neurological or psychiatric problems participated in the study for payment. All of the participants were naïve to tDCS, the trust game and the dictator game, had normal or corrected-to-normal vision, and gave their written informed consent, which was approved by the Zhejiang University ethics committee. The whole experiment lasted approximately 60 min, and each participant received a payment of around 46 RMB Yuan (approximately 7.012 US dollars) on average after finishing their tasks. No participants reported any adverse side effects concerning pain on the scalp or headaches after the experiment.

#### tDCS

In tDCS, a weak direct current is applied to the scalp via two saline-soaked surface sponge electrodes (35 cm^2^). The current was constant and was delivered by a battery-driven stimulator (NeuroConn, Germany). It was adjusted to induce cortical excitability of the target area without any physiological damage to the participants. Various configurations of the current had various effects on cortical excitability. Generally speaking, anodal stimulation enhances cortical excitability, whereas cathodal stimulation suppresses it (Nitsche and Paulus, 2000).

**FIGURE 1:**
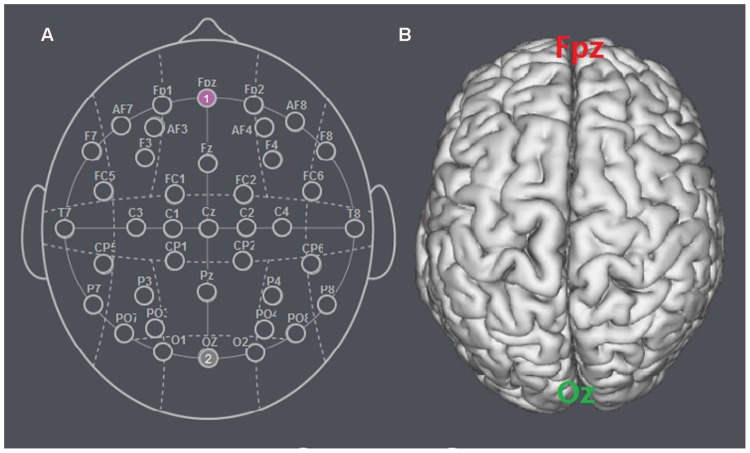
**Locations of the electrode positions. (A)** Schematic of the electrode positions Fpz and Oz based on the international EEG 10-20 system. **(B)** Locations of the vmPFC (Fpz) and the visual cortex (Oz) of the human brain.

**FIGURE 2:**
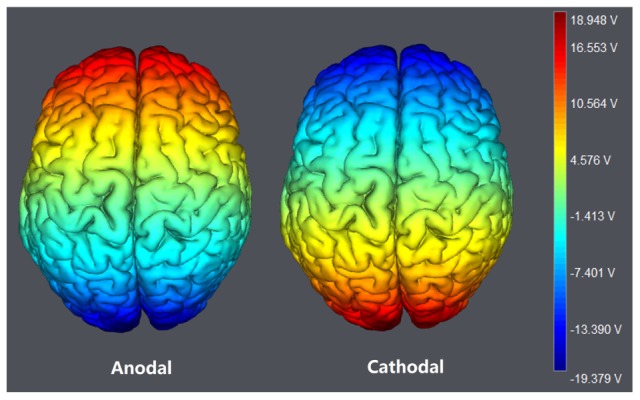
**The stimulation modes of tDCS treatments.** The axis represents the range of input voltage from -19.379 to 18.948 V.

The participants were randomly assigned to receive anodal tDCS over vmPFC (*n* = 20, 10 females), cathodal tDCS over vmPFC (*n* = 20, 10 females) or sham stimulation (*n* = 20, 11 females). For anodal stimulation, the anodal electrode was placed over vmPFC, at the Fpz position according to the international EEG 10/20 system, whereas the cathodal electrode was placed over Oz (Colzato et al., 2015). For cathodal stimulation, the cathodal electrode was placed over Fpz, whereas the anodal electrode was placed over Oz (**Figures 1** and **2**). For sham stimulation, the procedures were the same, but the stimulation was turned off after 30 s without the participants’ knowledge. The participants may have felt the initial itching, but there was actually no current for the rest of the stimulation. This method of sham stimulation has been shown to be reliable (Gandiga et al., 2006). The current was constant and of 2 mA in intensity, with a 30 s ramp up and down; the safety and efficiency of this stimulation has been demonstrated in previous studies. Before the decision making tasks, the laboratory assistant put a tDCS device on the participant’s head for stimulation. After 20 min of stimulation, the tDCS device was taken off and the participant was then asked to complete several economic interaction games.

#### Task and Procedure

**FIGURE 3:**
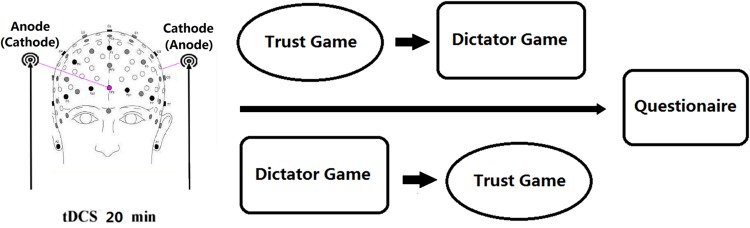
**Experimental design.** After 20 min of stimulation, each participant completed Trust Game and Dictator Game with anonymous others. Half of the participants completed the Trust Game first, while the rest completed the Dictator Game first.

After the participants received tDCS stimulation for 20 min (single-blinded, sham-controlled), they completed two economic decision tasks programmed by z-Tree (Fischbacher, 2007). To eliminate the sequence effect of the two tasks, we randomly assigned half of the participants to complete the trust game first, while the other half to complete the dictator game first (**Figure 3**). Because social interaction experiments such as the trust game, dictator game, public good game, and ultimatum game require the simultaneous interaction of a number of subjects, eliminating their suspicion of whether they are playing with real person which may alter their behaviors (Frohlich et al., 2001), we managed 10 participants in the same laboratory during an experimental session who were anonymous to each other in separated cubicles.

##### Trust game

The trust game followed the classical design originally performed by Berg et al. (1995). There are two roles in the trust game: trustor and trustee. Each role is offered a certain amount of original endowment (for example: 10 tokens). Firstly, the trustor decides the amount to transfer to the trustee. Then, the transferred amount is tripled, and the trustee should decide how much of the tripled amount to transfer back to the trustor. For example, if the amount being transferred by the trustor is X and the amount being transferred back by the trustee is Y, then the trustor will receive 10 - X + Y, and the trustee will receive 10 + 3X - Y.

In our Trust Game task, after our participants passed two profit-calculating questions (Supplementary Material) ensuring that they fully understood the trust game, each participant had to decide how much of the original endowment (ten tokens) to transfer to another participant player B (trustee) when he or she was playing the role of player A (trustor). Consequently, each participant was asked to estimate the amount sent back by her partner in each possible condition. We used the strategy method that has been proved reliable (Brandts and Charness, 2000; Ashraf et al., 2006), and each correct estimation (when the difference between the estimation and the partner’s choice is less than or equal to 1) was rewarded one extra token. Thirdly, our participants made decisions when playing the role of trustee. The trustee had to decide on a contingent action for every possible amount sent by the trustor. Such a role reversal design has also been demonstrated reliable (Charness and Rabin, 2002; Brandts and Charness, 2011). Our participants were informed about how their decisions determined their final payments: the game was played only once with each participant randomly paired with another participant and in the final stage of the experiment, the role he or she played in this game would also be randomly assigned by the computer.

##### Dictator game

We applied the dictator game originally designed by Forsythe et al. (1994) in our experiment. In the dictator game, there are two roles: dictator and receiver. The dictator has to divide a certain amount of money (for example: 10 tokens) between herself and the receiver, while the receiver can only accept the division. For example, if the dictator transfers X to the receiver, the dictator gets 10 - X, and the receiver gets X.

Firstly, after our participants passed one profit-calculating question (Supplementary Material) ensuring that they fully understood the game, each participant was asked how much to transfer to player B (receiver) when playing the role of player A (dictator). Consequently, when playing the receiver, each participant was asked to estimate the amount transferred by her partner in the role of dictator. Correct estimation (when the difference between the estimation and the partner’s choice is less than or equal to 1) was again rewarded one extra token. Our participants were also informed about how their decisions would determine their final payments: the game was played only once with each participant randomly paired with another participant, and in the final stage of the experiment, the role that he or she played in this game would be randomly assigned by the computer. In this case, our participants would never know who their partner was in each task. Each participant played four trials in total (two in the trust game as trustor and trustee, and two in the dictator game as dictator and receiver). Since there were 10 participants in one experimental session, five pairs of partnership were performed for each session. The decisions made by the computer for the trust game and the dictator game were independent which means that the partnerships and the roles played in the former game did not influence those in the latter game.

When all of the participants finished the two tasks, they were asked to complete a questionnaire before receiving their payments. Then, our participants were shown which roles they played in each game and received their payments according to their own choices and the choices of their partners. The exchange rate for game tokens and RMB Yuan was 1:1, and each participant received an extra 20 RMB Yuan for participation.

### Results

#### Behavioral Data

Behavioral data were statistically evaluated using SPSS software (version 22, SPSS Inc., Chicago, IL, USA). The significance level was set at 0.05 for all analyses. In the trust game, the amount transferred (trust investment) in the role of trustor indicates our participants’ trust, while the rate of amount sent back in the role of trustee indicates their trustworthiness. Notably, given a certain level of trust investment, the absolute repayment amount of the trustee does not represent a trustee’s trustworthiness level. It is reasonable to normalize the amount transferred back to the tripled investment amount. We defined the repayment rates of the transfer back to the tripled investment as Ratio that is a valid measurement of our participants’ trustworthiness. The trust investment of each participant is defined as SelfTrust (measuring trust). In the dictator game, the amount transferred in the role of dictator is defined as DGgive (measuring the altruistic preference).

First, we tested whether there is any sequence effect. No significant difference between the two different sequences in our sham group was observed either in the trustworthiness [*t*(18) = -0.594, *p* = 0.560, independent sample test] or in the altruistic preference [*t*(18) = 0, *p* = 1.000, independent sample test]. Although the sequences in our experiment were counterbalanced across tDCS stimulations, the trustworthiness and the altruism were also analyzed by means of analyses of variance (ANOVAs) with tDCS stimulation type and sequence as between-subjects factors. We observed neither a main effect of sequence (trustworthiness: *F* = 0.001, *p* = 0.975; altruism: *F* = 0.523, *p* = 0.473) nor interaction effects of stimulation type and sequence (trustworthiness: *F* = 0.266, *p* = 0.768; altruism: *F* = 0.154, *p* = 0.857).

**FIGURE 4:**
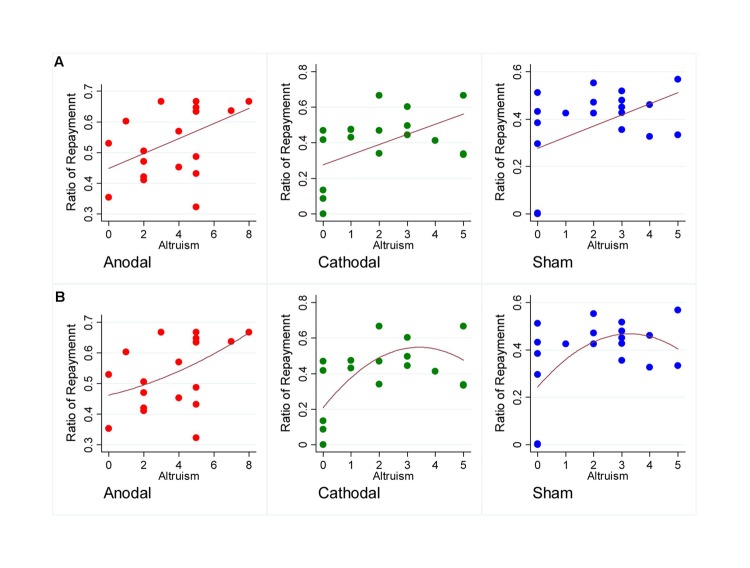
**Scatter plots of participants.** The horizontal axis represents the mean repayment rate and the vertical axis represents the altruistic preference. **(A)** The line of best fits for scatter plots of participants receiving different stimulations. **(B)** The quadratic curve of best fits for scatter plots of participants receiving different stimulations.

Secondly, results similar to previous studies were observed. The average Ratio in the trust game of each participant was significantly related with the DGgive in the dictator game in our sham group participants (Coefficient = 0.479, *p* = 0.033, Pearson correlation), and such a positive correlation was also found in the anodal group (Coefficient = 0.455, *p* = 0.044, Pearson correlation) as well as the cathodal group (Coefficient = 0.539, *p* = 0.014, Pearson correlation). The result revealed that the more altruistic participants tend to be more trustworthy in a trust game and such an observation was robust through all the three tDCS groups. It indicated that tDCS stimulation may have little influence over the correlation between trustworthiness and altruism. There is a steeper increasing trend of trustworthiness with the increase of altruistic preference in the line of best fit for anodal group comparing to those for cathodal and sham groups (see **Figure 4** for scatter plots and line of best fits). The quadratic curve of best fits may indicate that the relationship between trustworthiness and altruism seems tighter among participants with higher altruistic preference in anodal group than those in cathodal and sham groups.

#### Effects of tDCS Over vmPFC in Trust

Investment of the trustor (SelfTrust) in the trust game from the anodal and cathodal tDCS over vmPFC groups and the sham group were analyzed via analyses of variance (ANOVAs) with tDCS stimulation type (anodal, cathodal vs. sham) as a between-subjects factor. No significant effect of stimulation type was observed (*F* = 2.486, *p* = 0.092). *Post hoc* analyses (Bonferroni) showed that there were no significant differences either between the anodal group (mean = 6.30) and the sham group (mean = 4.65, *p* = 0.155) or between the cathodal group (mean = 4.75) and the sham group (mean = 4.65, *p* = 1.000).

#### Effects of tDCS Over vmPFC in Trustworthiness

**FIGURE 5:**
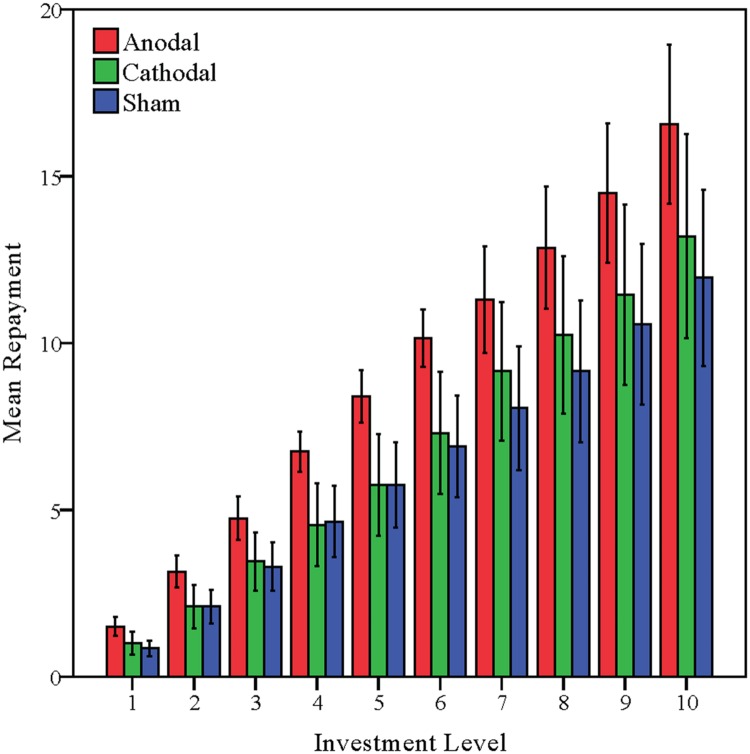
**Data of trustees’ repayment.** Mean repayment level across stimulations over the vmPFC. Error bars indicate 95% confidence intervals.

Ratios of the trustees in the trust game from the anodal and cathodal tDCS over vmPFC groups and the sham group were analyzed via repeated measures analyses of variance (ANOVAs) with investment level (possible investment level of partner) as a within-subjects factor and tDCS stimulation type (anodal, cathodal vs. sham) as a between-subjects factor. A significant influence of investment level was observed (*F* = 6.315, *p* = 0.003). There was an increasing trend of repayment level with the increase of the investment level (**Figure 5**). There was also a significant main effect of tDCS stimulation (*F* = 5.860, *p* = 0.005). The comparison of the mean Ratios of three tDCS groups is shown in **Table 1**. *Post hoc* analyses (Bonferroni) showed that the Ratios in the anodal group (mean = 0.536) were significantly higher than those obtained in the sham group (mean = 0.368, *p* = 0.007) or those obtained in the cathodal group (mean = 0.393, *p* = 0.027). No significant difference between the cathodal group and the sham group was observed (*p* = 1.000).

**Table 1:** Differences in trustworthiness among the anodal vmPFC, cathodal vmPFC, and sham groups (Ratio, %).

Stimulation	Investment Level									
Type	1	2	3	4	5	6	7	8	9	10
Anodal group	50.0	52.5	52.8	56.3	56.0	56.4	53.8	49.7	53.7	55.2
Cathodal group	33.3	35.0	38.3	37.9	38.3	40.6	43.6	39.4	42.4	44.0
Sham group	28.3	35.0	36.7	38.8	38.3	38.3	38.3	35.5	39.1	39.8

#### Effects of tDCS Over the vmPFC in Altruism

**FIGURE 6:**
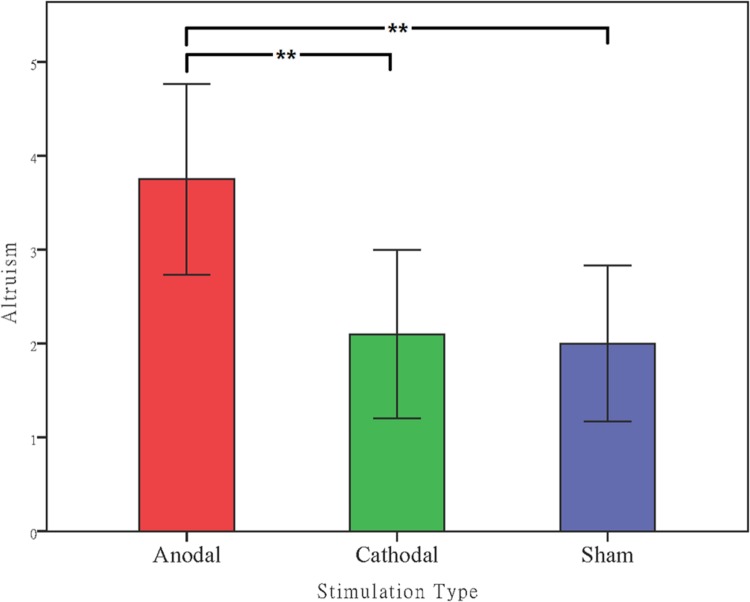
**Data of dictators’ transfer.** Mean altruistic transfer across stimulations over vmPFC. Error bars indicate 95% confidence intervals. Asterisks indicate statistical significance of difference between treatments.

The amounts transferred (DGgive) in the dictator game by the participants from the anodal and cathodal tDCS over vmPFC groups and the sham group were analyzed via analyses of variance (ANOVAs) with tDCS stimulation type (anodal, cathodal vs. sham) as a between-subjects factor. There was a significant main effect of tDCS stimulation (*F* = 5.015, *p* = 0.010). *Post hoc* analyses (Bonferroni) showed that the altruistic preference in the anodal group (mean = 3.75) was significantly higher than that obtained in the sham group (mean = 2.00, *p* = 0.020) and that obtained in the cathodal group (mean = 2.10, *p* = 0.030). No significant difference between the cathodal group and the sham group was observed (*p* = 1.000) (**Figure 6**).

### Discussion

Previous studies have revealed that trustworthiness is statistically correlated with the participant’s altruistic preference (Cox, 2004; Ashraf et al., 2006; Chaudhuri and Gangadharan, 2007). A similar pattern was observed for our sham group, confirming that altruism is closely correlated with trustworthiness. Those participants with higher levels of altruistic preference toward others were also more trustworthy in a trust game. The average ratios in the sham group also showed an increasing trend with the increase of the investment level (**Table 1**).

Neuroimaging studies have demonstrated that the vmPFC has special importance in human social cognition and behavior (Damasio, 1994; Anderson et al., 2006). Given that patients with vmPFC lesions show less trustworthiness behavior than control participants (Moretto et al., 2013), the irreplaceable function of this particular brain area has been demonstrated, whereas the influence of the vmPFC on the repayment ratios by the participants in the trust game has not been fully revealed. In the current study, we applied tDCS over the vmPFC in our participants to determine the influence of the vmPFC in trustworthiness. We found that enhancing the activation of the vmPFC significantly increased the repayment ratios in our participants compared to the sham group in the one-shot anonymous trust game, indicating that activating the vmPFC leads to more cooperative choices in trustees. There was no significant difference between the cathodal stimulation and the sham group. Our observations suggested a causal relationship between the activity of vmPFC and individuals’ trustworthiness, especially indicating that activating the vmPFC could lead to more cooperative behavior by trustees.

fMRI studies have shown that neural activity in the mPFC predicted greater empathy and altruistic motivation (Mathur et al., 2010; Waytz et al., 2012). Moreover, the finding that vmPFC patients were less altruism in the dictator game indicates that this brain region is closely related to empathy and altruistic behavior toward other individuals (Krajbich et al., 2009). However, the causal relationship between vmPFC and altruism preference has not been reported. In the current study, we observed that activation of the vmPFC by tDCS could significantly increase the altruistic behaviors of our participants in a dictator game, while no such significant effect of cathodal stimulation was observed.

Because convergent evidence has demonstrated that individuals with greater altruism and empathy are more trustworthy than those who have less empathy toward their partners, along with our observations that the anodal tDCS stimulation significantly increases altruistic preference as well as enhancing trustworthiness in tDCS experiments, we conclude that the vmPFC is closely associated with trustworthiness and altruism and that altering the activity of a brain region shared by those traits, such as the vmPFC, could significantly improve the cooperative behavior of trustees by enhancing their altruistic preferences.

Moreover, there is no significant effect of trust between different tDCS stimulations, which is consistent with another tDCS study (Colzato et al., 2015). We interpret this outcome as the result of various preferences underlying trust and trustworthiness. For instance, the behavior of trust is much more complicated than trustworthiness, such that many preferences including risk preference (Cook and Cooper, 2003; Schechter, 2007), altruism (Cox, 2004; Ashraf et al., 2006; Altmann et al., 2008), betrayal aversion (Bohnet and Zeckhauser, 2004; Bohnet et al., 2008) and ambiguity (Bourgeois-Gironde et al., 2012) would influence individuals’ interpersonal trust levels. Those different preferences have various neural bases, indicating that the behavior of trust may be performed by the cooperative work of several brain areas.

Although Wang et al. (2016) interpreted their observations as the effect of activating the orbitofrontal cortex rather than of inhibiting the dorsolateral prefrontal cortex (dlPFC), which also plays an essential role in economic decision making (Sanfey et al., 2003; Knoch et al., 2006; Knoch and Fehr, 2007; Spitzer et al., 2007), it is impossible to eliminate the effect of both those brain regions. In the current study, we placed one electrode over the target brain area while the other electrode over the occipital lobe, which has been proved irrelevant to trusting behaviors. In spite of this, our experimental design does not ensure us that the observations in Experiment 1 were completely due to the stimulation effects over vmPFC rather than the stimulation effects over the reference site (for example, visual cortex).

To further elucidate this issue, a control experiment was performed (Experiment 2) to rule out the influence of visual cortex. In Experiment 2, we tested two more groups with the target electrode placed over another position within the prefrontal cortex (right dlPFC) and the reference electrode placed over visual cortex. Then, the observations including trustworthiness and altruism from anodal and cathodal tDCS over dlPFC groups were compared with those from the sham group. If no significant differences were observed between the participants’ trustworthiness and altruism from the anodal and cathodal dlPFC groups and those from the sham group, we could conclude that the promotion of cooperative behaviors revealed by comparing the observations from participants receiving anodal tDCS over vmPFC with those from participants receiving sham stimulation could only be due to the anodal stimulation effect of the specific target (vmPFC) rather than to a cathodal stimulation effect of the visual cortex.

## Experiment 2

### Materials and Methods

#### Subjects

A new sample of 40 right-handed healthy subjects (mean age 21.55 years, ranging from 17 to 30 years; 21 females) with no history of neurological or psychiatric problems participated in the study for payment. As in Experiment 1, all participants were naïve to tDCS, the trust game and the dictator game, had normal or corrected-to-normal vision, and gave their written informed consent. Participants also received a written explanation of the tDCS and of any possible adverse effects, but no information about the type of stimulation or the experimental hypotheses. The protocol was approved by the Zhejiang University ethics committee.

#### Apparatus, Tasks, and Procedure

**FIGURE 7:**
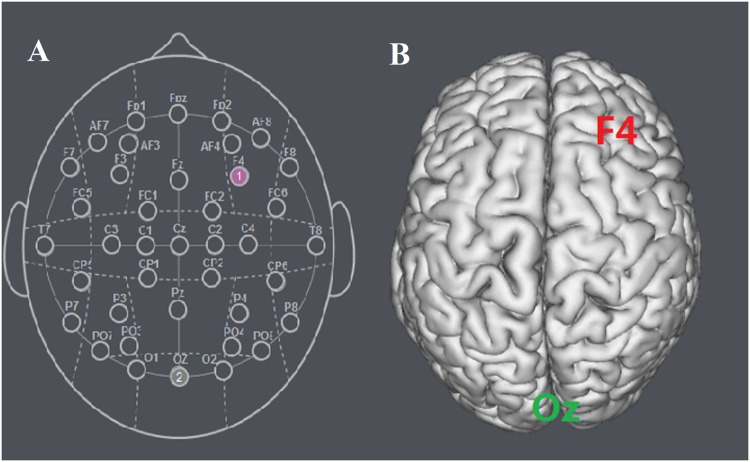
**Locations of the electrode positions. (A)** Schematic of the electrode positions F4 and Oz based on the international EEG 10-20 system. **(B)** Locations of the right dlPFC (F4) and the visual cortex (Oz) of the human brain.

The apparatus, tasks and procedure were exactly the same as that in the Experiment 1 with the following exception. Because the Experiment 2 served as a control to verify that the tDCS stimulation effects observed in Experiment 1 are specific to vmPFC, we tested two more groups with the target electrode placed over another position within the prefrontal cortex: the right dlPFC. The participants were randomly assigned to receive anodal tDCS over right dlPFC (*n* = 20, 11 females) or cathodal tDCS over right dlPFC (*n* = 20, 10 females). For anodal stimulation, the anodal electrode was placed over right dlPFC, at the F4 position according to the international EEG 10/20 system, whereas the cathodal electrode was placed over Oz. For cathodal stimulation, the electrodes were reversed (**Figure 7**).

### Results and Discussion

**FIGURE 8:**
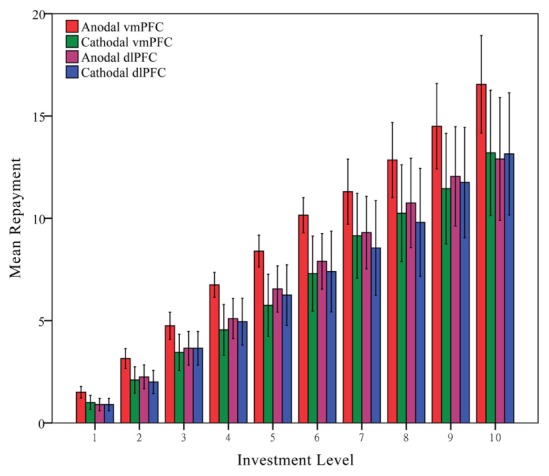
**Data of trustees’ repayment.** Mean repayment level across stimulations over the vmPFC and the right dlPFC. Error bars indicate 95% confidence intervals.

**Table 2** shows the comparison of the mean Ratios of three tDCS groups (anodal dlPFC, cathodal dlPFC, and sham groups). Given that Experiment 2 placed the tDCS stimulation over another position within the prefrontal cortex as a control site to verify that the enhancing tDCS effects in Experiment 1 was due to the stimulation over vmPFC, rather than the stimulation over visual cortex, observations measuring trustworthiness and altruism from the anodal dlPFC and cathodal dlPFC groups were compared with those from the sham group. As in Experiment 1, Ratios of the trustees in the trust game from the anodal and cathodal tDCS over dlPFC groups and the sham group were analyzed via repeated measures analyses of variance (ANOVAs) with investment level as a within-subjects factor and tDCS stimulation type as a between-subjects factor. Only a significant influence of investment level was observed (*F* = 20.662, *p* < 0.001), while no significant effect of stimulation type was observed (*F* = 0.278, *p* = 0.758). *Post hoc* analyses (Bonferroni) showed that there were no significant differences either between the anodal dlPFC group (mean = 0.411) and the sham group (mean = 0.368, *p* = 1.000) or between the cathodal dlPFC group (mean = 0.394) and the sham group (mean = 0.368, *p* = 1.000). We also compared Ratios of the trustees in the trust game from the anodal and cathodal tDCS over vmPFC groups and the anodal and cathodal tDCS over dlPFC groups via repeated measures analyses of variance (ANOVAs) with investment level as a within-subjects factor and tDCS stimulation type and stimulation site (vmPFC vs. dlPFC) as between-subjects factors. Similar results were observed and there were also a significant main effect of stimulation site (*F* = 3.912, *p* = 0.052) and significant interaction effects of stimulation site by stimulation type (*F* = 3.990, *p* = 0.049, **Figure 8**).

**Table 2:** Differences in trustworthiness among the anodal dlPFC, cathodal dlPFC, and sham groups (Ratio, %).

Stimulation	Investment Level									
Type	1	2	3	4	5	6	7	8	9	10
Anodal group	30.0	37.5	40.6	42.5	43.7	43.9	44.3	41.3	44.6	43.0
Cathodal group	30.0	33.3	40.6	41.3	41.7	41.1	40.7	37.7	43.5	43.8
Sham group	28.3	35.0	36.7	38.8	38.3	38.3	38.3	35.5	39.1	39.8

**FIGURE 9:**
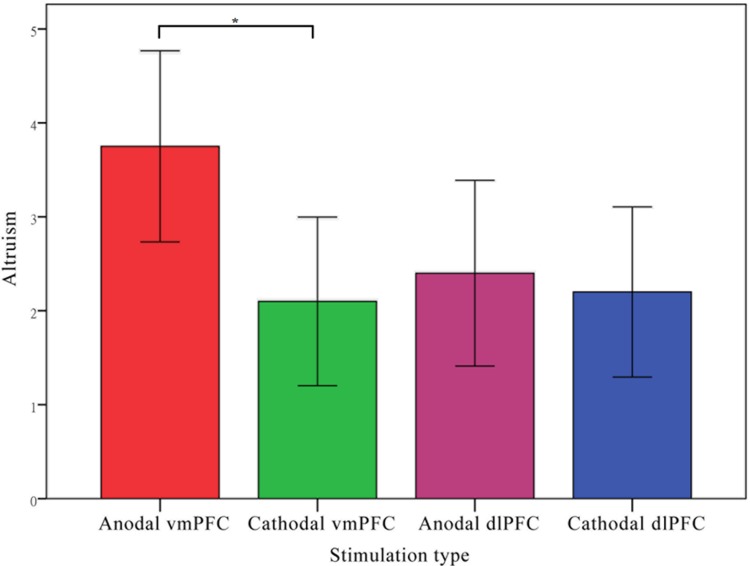
**Data of dictators’ transfer.** Mean altruistic transfer across stimulations over vmPFC and right dlPFC. Error bars indicate 95% confidence intervals. Asterisks indicate statistical significance of difference between treatments.

Moreover, as in Experiment 1, the amounts transferred (DGgive) in the dictator game by the participants from the anodal and cathodal tDCS over dlPFC groups and the sham group were analyzed via analyses of variance (ANOVAs) with tDCS stimulation type as a between-subjects factor. No significant main effect of tDCS stimulation was observed (*F* = 0.211, *p* = 0.810). *Post hoc* analyses (Bonferroni) showed that there were no significant differences either between the dlPFC anodal group (mean = 2.40) and the sham group (mean = 2.00, *p* = 1.000) or between the cathodal dlPFC group (mean = 2.20, *p* = 1.000) and the sham group. We also compared the amounts transferred (DGgive) in the dictator game from the anodal and cathodal tDCS over vmPFC groups and the anodal and cathodal tDCS over dlPFC groups via repeated measures analyses of variance (ANOVAs) with tDCS stimulation type and stimulation site (vmPFC vs. dlPFC) as between-subjects factors. There were also a significant main effect of stimulation site (*F* = 37.857, *p* < 0.001) and significant interaction effects of stimulation site by stimulation type (*F* = 7.626, *p* = 0.007, **Figure 9**).

The results of this experiment ruled out the possibility that the more cooperative behaviors observed in anodal vmPFC group of Experiment 1 were due to the stimulation effect over visual cortex rather than activating the excitability of vmPFC. Because previous studies have demonstrated that the right dlPFC is closely associated with repayment of trustees and because modulating the activity of that specific brain area alters the trustworthiness of participants in both tDCS and TMS studies (Knoch et al., 2009; Nihonsugi et al., 2015), it is reasonable to argue that the anodal tDCS effect over the vmPFC in Experiment 1 might, in fact, be due to anodal current traveling through cortices adjacent to the vmPFC (such as the dlPFC) rather than to altering the activity of vmPFC. To the extent that the enhancing effect of cooperative behaviors in Experiment 1, in which participants receiving anodal stimulation over the vmPFC were more trustworthy and altruistic than those receiving sham stimulation, was caused by anodal current traveling through the dlPFC, participants receiving anodal stimulation over the dlPFC in Experiment 2 should also significantly repay more as trustee and transfer more as dictator than would participants in the sham group. In fact, the observations in Experiment 2 provide straightforward evidence that the promoted cooperative behaviors observed in Experiment 1 were caused specifically by tDCS-induced changes in vmPFC activity.

Although the repayment ratios in the trust game and the transferred amount in the dictator game by individuals receiving anodal tDCS over the dlPFC were slightly higher than those receiving cathodal tDCS over the dlPFC and those receiving sham stimulation, no significant differences in trustworthiness and altruism between participants in the anodal dlPFC group and those in the sham group were observed. We interpret the inconsistency between our observations and previous studies as a result of measuring trustworthiness with different task procedures. Knoch et al. (2009) found that when the participant’s right dlPFC was disrupted by rTMS, the trustee failed to resist the temptation to defect in the trust game while playing the trust game for many periods. However, no significant difference was observed in their one-shot anonymous trust game. The tDCS study performed by Nihonsugi et al. (2015) also involves concern for reputation. Hence, the influence of dlPFC in altering the cooperative behaviors in the trust game might be due to external factors such as concern for reputation, and the results of Experiment 2 added further evidence to previous observations indicating that modulating the excitability of the right dlPFC by tDCS may not alter participants’ trustworthiness or altruistic preference in one-shot anonymous economic interactions.

Previous studies have also revealed that reducing the activity of the right dlPFC would decrease the rejection rates of unfair offers in the ultimatum game (Knoch et al., 2006, 2008), and these results were interpreted as the dlPFC directly suppressing neural activity that represented a self-interested impulse. We observed that activating the vmPFC leads to more cooperative behaviors in the trust game and the dictator game, while no such effect has been observed by modulating the cortical activity of the right dlPFC. This implies that the vmPFC may be specifically associated with decisions involving purely cooperative motives (such as increasing altruism), while the right dlPFC seems to be more closely correlated with considerations involving self-interested motives (such as reducing fairness preference and concern for reputation).

In addition, since the mechanism of tDCS is that anodal stimulation enhances cortical excitability, whereas cathodal stimulation suppresses it, typical anodal-excitation and cathodal-inhibition effects should be observed. However, in both Experiments 1 and 2 of our current study, relatively few significant cathodal-inhibition results have been revealed in comparison to anodal-excitation effects. Such an asymmetric stimulation effect also exists widely in previous tDCS studies, especially among cognitive or perceptual tasks. This issue has been discussed in depth by Jacobson et al. (2012), and they argued that the lack of inhibitory cathodal effects might reflect compensation processes because cognitive functions are typically supported by rich brain networks. More recently, Price et al. (2015) also reported that single-session tDCS, especially anodal stimulation produces significant effects on cognitive studies, which may add further evidence to this issue.

## Conclusion

In this study, we extended studies regarding the function of the vmPFC in altruism and trustworthiness behaviors, revealing that activating this neural region can promote the cooperative behaviors in both the dictator game and the trust game. Moreover, our finding demonstrates that anodal tDCS over the vmPFC could particularly alter the trustworthiness of trustees and their altruistic preference, but no significant influence over interpersonal trust was observed. Our observations indicate that other-regarding preferences such as altruism are closely correlated with trustworthiness and that modulating the activity of vmPFC could enhance trustworthiness of trustees through increasing their altruistic preference. In previous studies, the activity of vmPFC has been revealed associated with social preference (Zaki et al., 2013), valuation (Smith et al., 2010), emotion (Winecoff et al., 2013), and empathy (Janowski et al., 2013), etc. Our observations imply that the stimulation effect may be interpreted as affecting the cognitive function of vmPFC involving empathy and valuation, which have been considered as factors influencing altruistic behaviors. However, further studies are needed to separate these factors, respectively.

One limitation of the current study is that although our findings for the vmPFC were consistent with previous observations, the potential neural mechanism of the specific brain area influencing trustworthiness through altering our participants’ altruistic preference remains to be revealed and discussed. Future brain imaging studies may focus on the dynamic activation of the vmPFC while the participants play the trust game or the dictator game. Another deficiency of our study is that the reason why stimulating vmPFC alters trustworthiness and altruism without producing a similar effect on our participants’ interpersonal trust levels has not been fully demonstrated. Further neural imaging studies specifically focusing on the trust of individuals might answer this question.

In sum, our findings provide important information about the impact of tDCS on healthy participants, especially with respect to the trustworthiness of trustees and their altruism. Activating the vmPFC by tDCS can enhance the trustworthiness in the trust game, specifically through increasing the altruism of our participants, who transferred more in the dictator game. Moreover, the vmPFC is specific for the trustworthiness of trustees and the altruistic preference of the dictator, while no tDCS stimulation effect was observed in the trust of trustors.

## Author Contributions

HZ, DH, SC, SW, WG, JL, HY, and YC designed experiment; HZ, DH, SC, SW, WG, JL, HY, and YC performed experiment; HZ and SC analyzed data; HZ drew figures; HZ, DH, SC, JL, and YC wrote the manuscript; HZ, DH, SC, SW, WG, JL, HY, and YC revised the manuscript; and HZ, DH, SC, SW, WG, JL, HY, and YC finally approved the version to be published.

## Conflict of Interest Statement

The authors declare that the research was conducted in the absence of any commercial or financial relationships that could be construed as a potential conflict of interest.
