# Supplementary material for: Modulating the Activity of Ventromedial Prefrontal Cortex by Anodal tDCS Enhances the Trustee’s Repayment through Altruism
Source: Front Psychol. 2016 Sep 22;7:1437. doi: 10.3389/fpsyg.2016.01437 (PMC5031609; doi:10.3389/fpsyg.2016.01437)
Supplement: Supplementary file 1 [file Data_Sheet_1.DOC]

Profit calculating questions:

Trust game:

Question1:

“Now you are in game testing question stage. Only passing these testing questions correctly which means you fully understand the game can you enter the formal game. You should insert the correct answer in the blank below. For experiment 1, if you transfer 6 tokens as Player A to somebody else playing the role of Player B, and Player B repays you 8 tokens, how many tokens do you get in this case? ”

The correct answer is 12 tokens.

Question2:

“Now you are in game testing question stage. Only passing these testing questions correctly which means you fully understand the game can you enter the formal game. You should insert the correct answer in the blank below. For experiment 1, when you are playing the role of Player B, if somebody else playing the role of Player A transfers 6 tokens to you, and you repays him or her 8 tokens, how many tokens do you get in this case?”

The correct answer is 20 tokens.

Dictator game:

Question:

“Now you are in game testing question stage. Only passing these testing questions correctly which means you fully understand the game can you enter the formal game. You should insert the correct answer in the blank below. For experiment 2, if you transfer 6 tokens as Player A to somebody else playing the role of Player B, how many tokens do you get in this case?”

The correct answer is 4 tokens.
